# Maternal Characteristics Affect Fetal Growth Response in the Women First Preconception Nutrition Trial

**DOI:** 10.3390/nu11102534

**Published:** 2019-10-21

**Authors:** K Michael Hambidge, Carla M. Bann, Elizabeth M. McClure, Jamie E. Westcott, Ana Garcés, Lester Figueroa, Shivaprasad S. Goudar, Sangappa M. Dhaded, Omrana Pasha, Sumera A. Ali, Richard J. Derman, Robert L. Goldenberg, Marion Koso-Thomas, Manjunath S. Somannavar, Veena Herekar, Umber Khan, Nancy F. Krebs

**Affiliations:** 1 Section of Nutrition, Department of Pediatrics, University of Colorado School of Medicine, Aurora, CO 80045, USANancy.Krebs@cuanschutz.edu (N.F.K.); 2 RTI International, Durham, NC 27709, USA; cmb@rti.org (C.M.B.); mcclure@RTI.org (E.M.M.); 3 INCAP (Instituto de Nutrición de Centro América y Panamá), Guatemala City 01011, Guatemala; agarces@incap.int (A.G.); lfigueroa@incap.int (L.F.); 4 KLE Academy of Higher Education and Research’s Jawaharlal Nehru Medical College, Belagavi, Karnataka 590010, India; sgoudar@jnmc.edu (S.S.G.); drdhadedsm@gmail.com (S.M.D.); manjunathsomannavar@gmail.com (M.S.S.); nandhari@gmail.com (V.H.); 5 Department of Community Health Sciences, Aga Khan University, Karachi 74800, Pakistan; omrana.pasha@jhu.edu (O.P.); sumera.ali@aku.edu (S.A.A.); umber.s.khan@googlemail.com (U.K.); 6 Department of Global Affairs, Thomas Jefferson University, Philadelphia, PA 19107, USA; richard.derman@jefferson.edu; 7 Department of Obstetrics and Gynecology, Columbia University, New York, NY 10032, USA; rlg88@cumc.columbia.edu; 8 NICHD/NIH, Bethesda, MD 20852, USA; kosomari@mail.nih.gov

**Keywords:** nulliparous, newborn anthropometry, preconception, maternal nutrition

## Abstract

The objective of this secondary analysis was to identify maternal characteristics that modified the effect of maternal supplements on newborn size. Participants included 1465 maternal–newborn dyads in Guatemala, India, and Pakistan. Supplementation commenced before conception (Arm 1) or late 1st trimester (Arm 2); Arm 3 received usual care. Characteristics included body mass index (BMI), stature, anemia, age, education, socio-economic status (SES), parity, and newborn sex. Newborn outcomes were *z*-scores for length (LAZ), weight (WAZ), and weight to length ratio-for-age (WLRAZ). Mixed-effect regression models included treatment arm, effect modifier, and arm * effect modifier interaction as predictors, controlling for site, characteristics, and sex. Parity (para-0 vs. para ≥1), anemia (anemia/no anemia), and sex were significant effect modifiers. Effect size (95% CI) for Arm 1 vs. 3 was larger for para-0 vs. ≥1 for all outcomes (LAZ 0.56 (0.28, 0.84, *p* < 0.001); WAZ 0.45 (0.20, 0.07, *p* < 0.001); WLRAZ 0.52 (0.17, 0.88, *p* < 0.01) but only length for Arm 2 vs. 3. Corresponding effects for para ≥1 were >0.02. Arm 3 *z*-scores were all very low for para-0, but not para ≥1. Para-0 and anemia effect sizes for Arm 1 were > Arm 2 for WAZ and WLRAZ, but not LAZ. Arm 1 and 2 had higher WAZ for newborn boys vs. girls. Maternal nulliparity and anemia were associated with impaired fetal growth that was substantially improved by nutrition intervention, especially when commenced prior to conception.

## 1. Introduction

Maternal characteristics have a major role in determining placental function and consequently fetal growth [1]. Notable among these characteristics are maternal anthropometric measures of malnutrition such as underweight [2,3]. Others include parity, age, socio-economic status (SES), education, stress, inflammation, anemia, and reduced utero–placental blood flow [1]. All of these and other environmental factors may affect the placental, and hence the fetal, supply of nutrients either by impacting maternal nutrition or/and by reducing utero–placental blood flow. In low-resource populations, dietary diversity and quantity are frequently limited, and maternal anthropometric indicators of both short- and long-term under-nutrition are common [4,5]. In these circumstances, the available maternal nutrient supply to the placenta is also likely to be the primary factor determining the placental and fetal supply of nutrients. Nutritional inadequacy in utero may result in a ‘fetal programming effect’ with longer-term effects on offspring growth [3,6]. Maternal anthropometric measures in these populations are strongly associated with fetal growth assessed by measures of newborn size [7]. Specifically, maternal underweight is associated with multiple measures of newborn size including low birth weight. Short maternal stature also increases the risk of low birth weight and small-for-gestational-age (SGA) infants [8,9,10,11]. Maternal anemia is associated with higher risks of low birth weight and preterm birth [12]. A strong association between maternal parity and adverse newborn outcomes, including impaired fetal growth, has been well recognized in populations in Asia, Africa, and Latin America [13], with the highest odds of adverse outcomes being for nulliparous women <18 years of age. Socio-economic status (SES) also has a direct association with infant growth at one year but may only have an indirect effect on birth length through maternal anthropometry [14]. Maternal education is strongly associated with socio-economic status and newborn size [15].

However, there is less information on whether baseline maternal characteristics can modify the effects of nutrition supplements. One relatively recent meta-analysis of the effects of maternal gestational multi-micronutrient supplements in undernourished populations documented greater birth weight increases with higher maternal baseline BMI in 11 out of 12 studies [16]. An even more recent pooled analysis for 12 trials of maternal multi micronutrients in low-income sites revealed very similar results for maternal BMI <18.5 kg/m^2^ vs. BMI ≥18.5 kg/m^2^, with modest reductions in relative risks for low birth weight for both subgroups [17]. Secondary analyses for the preconception food supplement trial in the slums of Mumbai indicated a progressively greater decrease in the relative risk of low birth weight with increasing maternal BMI [18] but this contrasted, for example, with effects observed with trials of protein-energy supplements [19,20]. As with maternal anthropometry, there are more data for the association of maternal anemia with newborn size than for maternal baseline anemia as a moderator of the effect of maternal nutrition supplements on fetal growth.

Similarly, documentation of the association between parity and the changes in newborn anthropometry in response to maternal nutrition interventions has been less consistent among trials. A meta-analysis including 15 trials of multi micronutrients revealed only a small pooled effect of parity on reduction of newborn low birth weight or SGA with the effect for nulliparous women slightly larger than multiparous women [17]. In a maternal food-based trial commencing preconception, mean birthweight was substantially higher for parous than for nulliparous mothers receiving food supplements [18]. The effects of young maternal age may have collinearity with those of nulliparity but do not explain the effects of the latter [21]. The effects of parity on the newborn outcomes of mothers receiving lipid-based nutrition supplements or multiple micronutrients plus a high lipid food have been quite variable. Trials in Bangladesh and Malawi have revealed no association between parity and newborn anthropometric outcomes [22,23]; however, overall effect sizes were negative or very modest in these studies. A trial in Burkina Faso found a greater effect of the maternal supplements for multiparous women [24]. The combined parity effects of maternal lipid nutrient supplement for a comparable study in Ghana were equally modest; however, the pre-designated nulliparous subgroup had major effect sizes from the maternal nutrition supplement for birth weight and length [25].

A primary focus of the Women First Preconception trial was on changes in newborn anthropometry associated with maternal nutrition supplements, especially with those commenced prior to conception [26]. Also reported have been newborn outcome data limited to those participants in the three Women First sites who had gestational age determined by first trimester ultrasound [26,27,28,29]. Maternal underweight is common in two of these three Women First sites included in this paper, specifically in those located in India and Pakistan [5]. Maternal stunting is outstandingly common in the Guatemala site [30]. Maternal stunting rates also remain high in the sites located in S. Asia where the combination of maternal stunting and underweight aggravates the risk of retarded fetal growth [5,27,29]. Maternal anemia is also a major concern in S. Asia. The purpose of this secondary analysis was to determine the extent to which maternal baseline characteristics modified the newborn length and weight responses to the interventions in the Women First Preconception trial.

## 2. Materials and Methods

### 2.1. Study Design

This study was a secondary analysis of data from the Women First Preconception trial [26,31]. In this trial, participants were randomized to one of three arms. Arm 1 commenced the nutrition intervention at least 3 months prior to conception; Arm 2 received the same intervention commencing at the end of the first trimester; Arm 3 (control) received no trial supplement. The participants were women of childbearing age (16–35 years) who were members of low-resource, small-town, rural communities in Chimaltenango, Guatemala, Thatta, Sindh Province, Pakistan, and Belagavi, Karnataka, India. Those enrolled were all expecting to conceive within the following eighteen months and had screening hemoglobin (Hb) >8 g/dL. Apart from the screening Hb, participating women were unselected with respect to their long- or shorter-term nutritional status as determined by stature and BMI. Included in the current analysis were the maternal–newborn dyads who had gestational age determined from crown-rump length measurements of gestational age in the first trimester and had newborn anthropometric measures completed within 48 h of delivery [26] Maternal characteristics evaluated were maternal stature, BMI, anemia status at baseline (defined as Hb <12 g/dL), parity, age, SES, and education. Newborn sex was also included in the regression analysis. Newborn outcome measures were length-for-age *z*-score (LAZ), weight-for-age *z*-score (WAZ), and weight to length ratio-for-age *z*-score (WLRAZ) based on INTERGROWTH-21st fetal growth standards [32,33].

Differences were documented by trial arms, and effect sizes were compared for Arm 1 vs. Arm 3; Arm 2 vs. Arm 3; and Arm 1 vs. Arm 2. The three Women First sites with first trimester measurements of gestational age were included in these analyses: Guatemala, India, and Pakistan [26]. Recruitment and enrollment began in December 2013 and the final delivery occurred in March 2017.

### 2.2. Ethical Approval

The project was approved by the Colorado Multiple Institutional Review Board, University of Colorado, the local or/and national ethics committees for each of the three research sites: Guatemala—Comite de Etica Universidad Francisco Marroquin 034-14; India—Institutional Ethics Committee on Human Subjects Research, KLE Society’s JNMC Institutional Ethics Committee on Human Subjects Research MDC/IECHSR/2013-14/A25; Pakistan—Aga Khan University Ethical Review Committee 2753-CHS-ERC-13. Each board was registered with US Office of Human Research Protection and had Federal-wide Assurance in place. Written informed consent was obtained from all participants prior to study participation. The study protocol is available online: https://www.ncbi.nlm.nih.gov/pmc/articles/PMC4000057/.

### 2.3. Statistical Analyses

Mixed-effect regression models of neonatal anthropometry (i.e., *z*-scores of weight, length, and weight to length ratio) were conducted using SAS PROC GLIMMIX (SAS, Cary, NC, USA). Models included treatment arm, effect modifier, and a treatment arm by effect modifier interaction to test for differential intervention impact. Each model included study site and cluster, which were included as random effects. The following variables were included as control variables: nulliparity, no formal education, age <20 years, low maternal body mass index (BMI) (BMI <18.5 kg/m^2^), anemia (Hb <12 g/dL), low SES, and newborn sex. Maternal stature had no effect on the final model. Analyses were conducted for each site individually and with the sites combined. Data described in the manuscript will be made publicly and freely available upon request without restriction at the National Institute of Child Health and Human Development Data and Specimen Hub (DASH, accession number pending).

## 3. Results

One thousand four hundred and sixty-five maternal–newborn dyads were included in this analysis (Figure S1 Consort Diagram). Of the potential effect modifiers listed in Table 1, maternal nulliparity and anemia emerged as significant maternal characteristic effect modifiers for the newborn outcomes for combined sites.

The distribution of nulliparous women varied by site with a very low percentage for Guatemala (Table 1). Nulliparous women had higher baseline percentages of age <20 years, no formal education, low BMI, anemia, and low SES. The percentage stunted was lower in nulliparous subjects and their mean height was higher, though mean weight was lower (Table 2).

**Figure 1:**
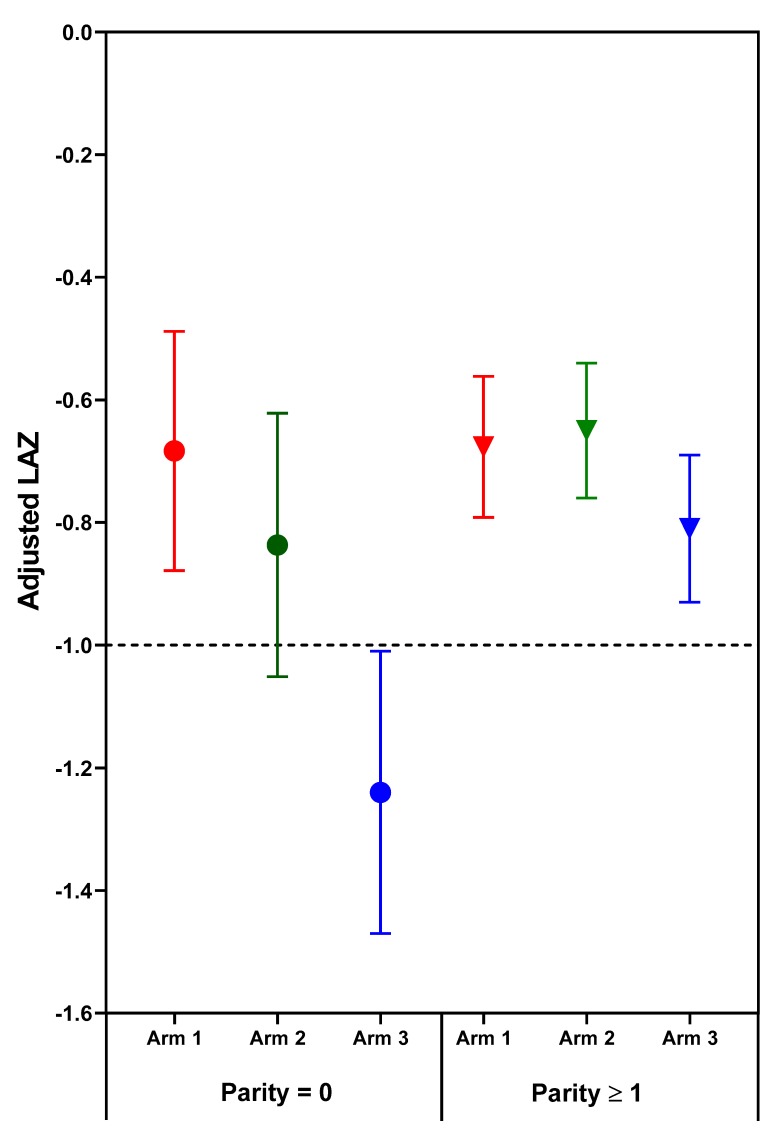
Adjusted mean (95% confidence interval) length-for-age *z*-scores (LAZ) of neonatal outcomes by treatment arm and parity. Horizontal line at LAZ −1 depicts a length deficit that has predicted a high risk of stunting at two years. Circles indicate parity = 0; Triangles indicate parity ≥1.

**Figure 2:**
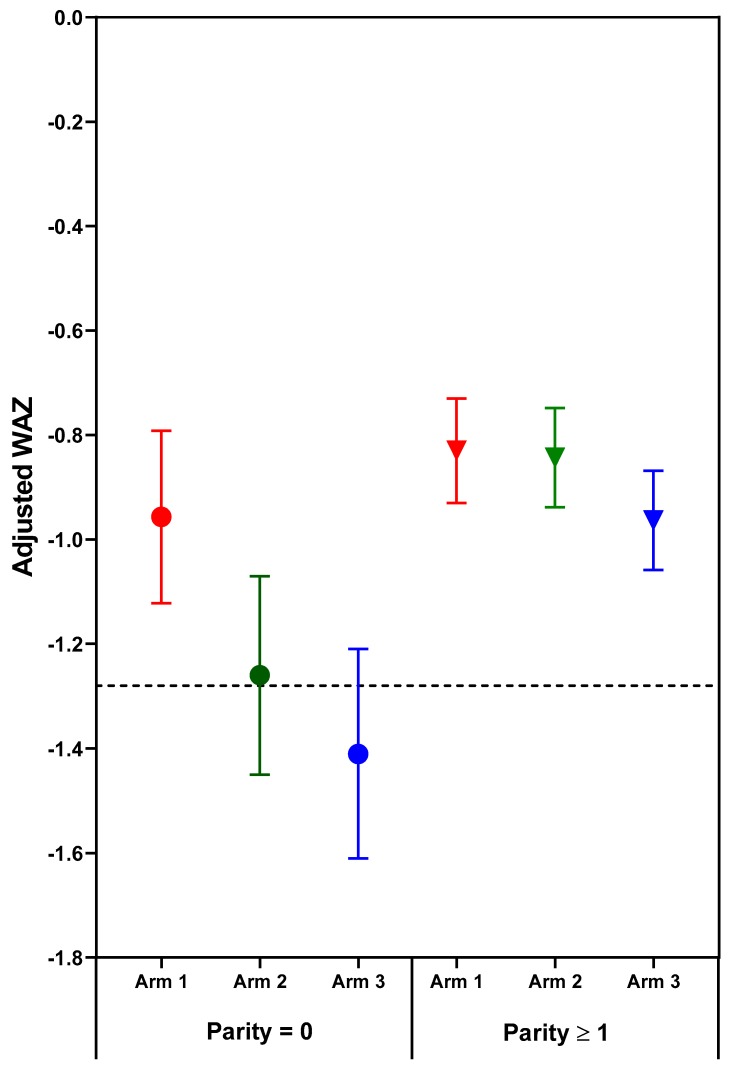
Adjusted mean (95% confidence interval) weight-for-age *z*-scores (WAZ) of neonatal outcomes by treatment arm and parity. Horizontal line at WAZ −1.28 corresponds to the 10th% WAZ (small-for-gestational age). Circles indicate parity = 0; Triangles indicate parity ≥1.

**Figure 3:**
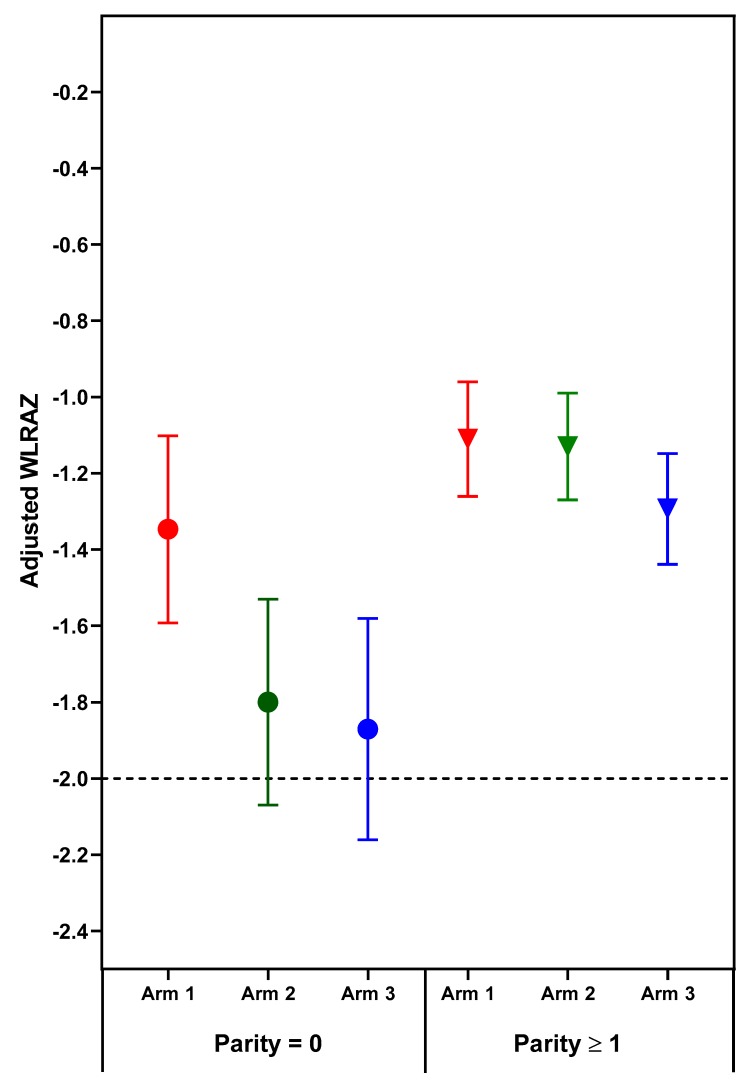
Adjusted mean (95% confidence interval) weight to length ratio-for-age *z*-scores (WLRAZ) of neonatal outcomes by treatment arm and parity. Horizontal line at WLRAZ −2 depicts weight to length deficit for diagnosis of wasting. Circles indicate parity = 0; Triangles indicate parity ≥1.

The effect of treatment on newborn length-for-age and weight-for-age depended on parity. Significant (*p* < 0.05) treatment arm x parity interactions for length- and weight-for-age *z*-scores (*p* = 0.031 and *p* = 0.038, respectively) were found for combined sites (Table 3, Figure 1 and Figure 2). Among nulliparous women, those in Arm 1 or 2 had newborns with significantly longer length than those receiving usual care (Arm 3). Arm 1 was associated with higher infant birth weights than either Arm 2 or 3; no significant differences were found for parous women. Although the treatment x parity interaction was not statistically significant at *p* < 0.05 for weight to length ratio-for-age *z*-score (*p* = 0.064), the pattern of differences appears to be similar to the findings for weight, with nulliparous women in Arm 1 having newborns with higher weight- and weight to length ratio-for-age *z*-scores than those in either Arm 2 or 3 (Table 3, Figure 2 and Figure 3). In subgroup analyses of nulliparous subjects alone, low baseline BMI vs. normal/high BMI was associated with more favorable effect sizes (Table S1. Descriptive subgroup analysis by parity and BMI). For nulliparous women in individual sites, the largest adjusted mean differences were for Arm 1 vs. Arm 3. (Table 4).

Treatment arm x maternal baseline anemia (Hb <12 g/dL) interactions were significant for all three outcomes (Table 5). Compared with usual care (Arm 3), both Arms 1 and 2 were associated with significantly higher infant length-, weight-, and weight to length ratio-for-age *z*-scores among women with anemia; Arm 1 had a larger effect than Arm 2 for WAZ (*p* = 0.028) and WLRAZ (*p* = 0.022). No significant differences were observed across treatment arms among women who were not anemic. For individual sites, effect moderator (maternal anemia at baseline) x treatment arm interactions were present for LAZ in Pakistan and for all three outcomes in Guatemala with corresponding significant effect sizes for Arm 1 vs. 3 (Table 6).

Treatment effects also varied significantly by newborn sex for newborn weight (*p* = 0.028) with marginally significant results for weight to length ratio-for-age *z*-score (*p* = 0.052) and length-for-age *z*-score (*p* = 0.071). Male newborns of women in Arms 1 and 2 had better outcomes for LAZ and WAZ than male newborns of control mothers (Arm 3). No significant treatment effects were observed for female newborn (Table S2. Regression results of neonatal anthropometry by newborn sex: combined sites). Newborn sex data were included in the final model.

No other maternal characteristics included in Table 1 exhibited significant moderator x treatment arm interactions. However, data for these characteristics were included in the final regression model.

## 4. Discussion

Of the maternal characteristics examined, nulliparity and maternal anemia at baseline were the only significant effect modifiers when controlled for all other potential modifiers examined and for each other. Positive effects for newborn length-, weight-, and weight to length ratio-for-age *z*-score were greatest for nulliparous women who started nutrition supplements at least three months prior to conception (Arm 1 vs. 3). For Arm 1 newborns of nulliparous women, the LAZ was in excess of ½ SD higher than for Arm 3. For weight- and weight to length ratio-for-age *z*-scores ≥ −1, these positive effects were also greater for the preconception arm compared to the arm commencing the same supplements in the first trimester of pregnancy (Arm 1 vs. 2). Details of the interactions between nulliparity and treatment arm did vary by site. For India, this interaction was significant for weight to length ratio-for-age *z*-scores and for Pakistan for LAZ. The percentage of <20 years of age was much greater among nulliparous than among parous women and the prevalence of maternal undernutrition (BMI <18.5 kg/m^2^) was higher in the nulliparous than parous women. However, neither the interaction of maternal age nor BMI x treatment arm was significant, though the addition of maternal BMI (but not maternal stature) had a minor impact on the final regression model.

The presence of maternal anemia at baseline modified the treatment effect for all three outcomes. For weight and weight to length ratio, these improvements were significantly greater for Arm 1 (commencing prior to conception) vs. Arm 2 (commencing early in gestation). Our results were reminiscent of those for a lipid nutrient supplement plus multiple micronutrient trial in rural Burkina Faso [24], except in that trial, the benefits of the supplement were only apparent for parous women. As in that trial, no treatment effect on birth length or weight was found for non-anemic mothers. In both studies, the interventions provided only 20 mg iron/day in contrast to a pregnancy trial of lipid nutrition supplements in Ghana in which a control arm received 60 mg iron daily [25]. In that study, maternal Hb and iron status were lower in the treatment arms [34].

The deficit in newborn length and weight for the offspring of nulliparous control mothers (Arm 3) is much greater than the minor deficits in newborn length and weight for the parous control mothers. Moreover, the greater vulnerability of fetal growth for nulliparous women was highly responsive to the nutrition intervention, especially the preconception arm. One potentially relevant relationship that has emerged from the Women First data is that that maternal nutrition supplements are most effective and perhaps only effective when fetal growth is quite severely compromised. Two indicators of impaired fetal growth responsive to maternal nutrition supplements are nulliparity and maternal baseline anemia. This relationship is illustrated in Figure 1, Figure 2 and Figure 3, in which the deficits in newborn *z*-scores are much greater for the nulliparous women in Arm 3 (controls) than for the parous women in Arm 3. The relatively large effect sizes for LAZ in Arm 1 and 2, especially the former, for the nulliparous women reduced the mean deficits to better than −1 *z*-score for LAZ, a deficit which portends a relatively high risk of stunting at 2 years of age [35]. Likewise, the effects of the preconception intervention were sufficient to improve the mean birth weight above that for small-for-gestational age, a well-established risk factor for stunting at 2 years of age [36].

Though maternal nulliparity and anemia have both been found to be maternal phenotypes alerting to the high risk of fetal growth retardation and to the substantial benefits of early attention to adequate nutrition, this does not preclude potential fetal benefits from early attention to maternal nutrition for women who do not have these phenotypes. The failure of maternal nutrition supplements to benefit the relatively small deficits in LAZ and WAZ at birth for parous and non-anemic (prior to conception) subjects in this cohort suggest that these relatively minor deficits may not be responsive to improvement in maternal nutrition, and additional environmental factors should be considered. In this regard, it is noted that the maximal improvements in these *z*-scores for nulliparous subjects with maternal nutrition supplements commenced prior to conception did not result in higher mean *z*-scores than those of the parous women (Figure 3). Both were comparable to mean deficits in *z*-scores for resource poor populations globally [37,38].

Weight to length ratio-for-age *z*-score is one continuous newborn outcome measure for Arm 1, in which the mean for nulliparous women did not improve to a value better than minus one. Though the magnitude of the improvement in WLRAZ for Arm 1 was comparable to that for LAZ and WAZ, the *z*-score deficit for Arm 3 was greater. Indeed, mean WLRAZ at −2 for Arms 2 and 3 corresponds to the cut-off for wasting. These low mean WLRAZ, even for Arm 1, suggest that further improvement in the duration, quality, or quantity of maternal supplements may still confer additional benefits on fetal growth. In this trial, further improvement in WLRAZ would be of special value in India where this *z*-score was outstandingly low. A corollary of this discussion is that a null or limited response to maternal nutrition interventions may be attributable to the population having only minor deficits at birth and thus having limited capacity to respond.

A weakness of this secondary analysis is the omission of the Democratic Republic of the Congo site for which gestational age data are not available. For this analysis, we concluded that the value of gestational age outweighed the inclusion of a fourth site. A further weakness is the paucity of nulliparous data for the site in Guatemala, where the relationship of newborn measures of fetal growth to maternal baseline anemia indicates that at least a subgroup of participants benefited from the maternal nutrition supplements.

Positive aspects of this analysis included the identification of maternal subgroups for which fetal growth benefited from the maternal nutrition supplements and clearly documented the considerable extent to which deficits in fetal growth can be corrected by improved maternal nutrition, especially if fetal growth deficits are relatively severe and improvements are commenced for a substantial period prior to conception. These results reinforce the need for more extensive nutrition research, policies, and interventions directed to women of childbearing age who are not mothers, with special attention to nulliparous women [39,40] and to women with anemia.

## 5. Conclusions

Maternal characteristics, as exemplified by nulliparity and by anemia in this randomized controlled trial, can have a major effect on fetal growth as measured with newborn anthropometry. A common characteristic of newborns who benefitted from maternal nutrition interventions is the severity of fetal growth deficits that, at least in this trial, could be predicted by nulliparity and by maternal anemia. The results of this secondary analysis provided further weight to the conclusion that maternal nutrition interventions are more effective in terms of birth weight if commenced sometime prior to conception.

## Figures and Tables

**Table 1:** Maternal characteristics, newborn sex, and risk factors by site.

Characteristic	All (*n* = 1465)	Guatemala (*n* = 493)	India (*n* = 515)	Pakistan (*n* = 457)
Maternal age, years	23.2 ± 4.1 ^1^	24.2 ± 4.4	22.0 ± 3.4	23.6 ± 4.1
20	296 (20) ^2^	75 (15)	131 (25)	90 (20)
20+	1169 (80)	418 (85)	384 (75)	367 (80)
Maternal stature, cm	149.6 ± 6.5	145.5 ± 4.8	151.2 ± 5.8	152.1 ± 6.6
Stunted ^3^	737 (50)	405 (82)	200 (39)	132 (29)
Not stunted	727 (50)	87 (18)	315 (61)	325 (71)
Maternal weight, kg	48.3 ± 9.3	53.7 ± 9.7	45.7 ± 8.0	45.5 ± 7.4
Maternal body mass index (BMI)	21.7 ± 4.4	25.3 ± 4.2	19.9 ± 3.2	19.7 ± 3.0
Low BMI ^4^	368 (25)	4 (1)	196 (38)	168 (37)
Normal/high BMI	1096 (75)	488 (99)	319 (62)	289 (63)
Parity				
=0	304 (21)	31 (6)	132 (26)	141 (31)
≥1	1161 (79)	462 (94)	383 (74)	316 (69)
Baseline hemoglobin (Hb)				
Anemia (Hb <12 g/dL)	855 (59)	59 (12)	470 (91)	326 (72)
No anemia (Hb ≥12 g/dL)	595 (41)	422 (88)	44 (9)	129 (28)
Household SES ^5^				
Low SES	315 (22)	52 (11)	49 (10)	214 (47)
Average/high SES	1150 (79)	441 (89)	466 (90)	243 (53)
Maternal education				
No formal education	461 (31)	35 (7)	40 (8)	386 (84)
Formal education	1004 (69)	458 (93)	475 (92)	71 (16)
Newborn sex				
Male	723 (49)	249 (51)	263 (51)	211 (46)
Female	742 (51)	244 (49)	252 (49)	246 (54)

^1^ Mean ± SD (all such values). ^2^ *n* (%) (all such values). ^3^ Stunted defined as <−2 SD height-for-age *z*-scores (HAZ). This is 147.9 cm for 15 years, 148.9 cm for 16 years, 149.5 cm for 17 years, 149.8 cm for 18 years and 150 cm for 19+ years. ^4^ Low BMI defined as <18.5 kg/m^2^. ^5^ The socio-economic status (SES) tally provides the number of indicators available from the following list: electricity, improved water source, sanitation, man-made flooring, improved cooking fuels, and household assets. Low SES is defined as having ≤2 SES indicators present.

**Table 2:** Maternal characteristics, newborn sex, and risk factors by parity and anemia for combined sites.

Characteristic	All (*n* = 1465)	Parity = 0 (*n* = 304)	Parity ≥1 (*n* = 1161)	*p*-Value	Anemia (*n* = 855)	No Anemia (*n* = 595)	*p*-Value
Maternal age, years	23.2 ± 4.1 ^1^	20.2 ± 3.2	24.0 ± 3.9	<0.001	22.7 ± 3.9	23.9 ± 4.3	< 0.001
<20	296 (20) ^2^	153 (50)	143 (12)	<0.001	195 (23)	99 (17)	0.004
20+	1169 (80)	151 (50)	1018 (88)		660 (77)	496 (83)	
Maternal stature, cm	149.6 ± 6.5	150.8 ± 6.2	149.3 ± 6.5	<0.001	151.1 ± 6.3	147.5 ± 6.2	< 0.001
Stunted ^3^	737 (50)	120 (39)	617 (53)	<0.001	332 (39)	394 (66)	< 0.001
Not stunted	727 (50)	184 (61)	543 (47)		523 (61)	201 (34)	
Maternal weight, kg	48.3 ± 9.3	46.3 ± 8.4	48.9 ± 9.4	<0.001	45.9 ± 8.0	51.7 ± 10.0	< 0.001
Maternal body mass index (BMI)	21.7 ± 4.4	20.4 ± 3.7	22.0 ± 4.5	<0.001	20.1 ± 3.4	23.8 ± 4.7	< 0.001
Low BMI ^4^	368 (25)	108 (36)	260 (22)	<0.001	295 (35)	72 (12)	< 0.001
Normal/high BMI	1096 (75)	196 (64)	900 (78)		560 (66)	523 (88)	
Baseline hemoglobin (Hb)							
Anemia (Hb <12 g/dL)	855 (59)	210 (69)	645 (56)	<0.001			
No anemia (Hb ≥12 g/dL)	595 (41)	93 (31)	502 (44)				
Parity							
Parity = 0					210 (25)	93 (16)	<0.001
Parity ≥1					645 (75)	502 (84)	
Household SES ^5^							
Low SES	315 (22)	77 (25)	238 (21)	0.068	205 (24)	108 (18)	0.008
Average/high SES	1150 (79)	227 (75)	923 (80)		650 (76)	487 (82)	
Maternal education							
No formal education	461 (31)	129 (42)	332 (29)	<0.001	324 (38)	135 (23)	<0.001
Formal education	1004 (69)	175 (58)	829 (71)		531 (62)	460 (77)	
Newborn sex							
Male	723 (49)	151 (50)	572 (49)	0.900	419 (49)	296 (50)	0.781
Female	742 (51)	153 (50)	589 (51)		436 (51)	299 (50)	

**Groups were compared using *chi*-square tests for categorical variables and *t*-tests for continuous variables.**^1^ Mean ± SD (all such values). ^2^ *n* (%) (all such values). ^3^ Stunted defined as <−2 SD height-for-age *z*-scores. This is 147.9 cm for 15 years, 148.9 cm for 16 years, 149.5 cm for 17 years, 149.8 cm for 18 years and 150 cm for 19+ years. ^4^ Low BMI defined as <18.5 kg/m^2^. ^5^ The socio-economic status (SES) tally provides the number of indicators available from the following list: electricity, improved water source, sanitation, man-made flooring, improved cooking fuels, and household assets. Low SES is defined as having ≤2 SES indicators present.

**Table 3:** Regression results of neonatal anthropometry by parity: combined sites.

	Arm 1	Arm 2	Arm 3	Parity x Arm	Arm 1 vs. Arm 3		Arm 2 vs. Arm 3		Arm 1 vs. Arm 2	
Outcome	Adjusted Mean (95% CI)	Adjusted Mean (95% CI)	Adjusted Mean (95% CI)	Interaction (*p*-Value)	Adjusted Mean Difference (95% CI)	*p*-Value	Adjusted Mean Difference (95% CI)	*p*-Value	Adjusted Mean Difference (95% CI)	*p*-Value
**Length-for-age *z*-score**										
Parity ≥1	−0.68 (−0.79, −0.56)	−0.65 (−0.76, −0.54)	−0.81 (−0.93, −0.69)	0.031	0.13 (−0.01, 0.28)	0.072	0.16 (0.02, 0.30)	0.024	−0.03 (−0.17, 0.11)	0.691
Parity = 0	−0.68 (−0.88, −0.49)	−0.84 (−1.05, −0.62)	−1.24 (−1.47, −1.01)		0.56 (0.28, 0.84)	<0.001	0.40 (0.11, 0.69)	0.007	0.16 (−0.11, 0.43)	0.254
**Weight-for-age *z*-score**										
Parity ≥1	−0.83 (−0.93, −0.73)	−0.84 (−0.94, −0.75)	−0.96 (−1.06, −0.87)	0.038	0.13 (0.00, 0.26)	0.046	0.12 (−0.00, 0.25)	0.056	0.01 (−0.12, 0.14)	0.883
Parity = 0	−0.96 (−1.12, −0.79)	−1.26 (−1.45, −1.07)	−1.41 (−1.61, −1.21)		0.45 (0.20, 0.70)	<0.001	0.15 (−0.12, 0.41)	0.273	0.30 (0.06, 0.54)	0.013
**Weight to length ratio-for-age *z*-score**										
Parity ≥1	−1.11 (−1.26, −0.96)	−1.13 (−1.27, −0.99)	−1.29 (−1.44, −1.15)	0.064	0.18 (0.00, 0.37)	0.047	0.17 (−0.01, 0.34)	0.066	0.02 (−0.16, 0.20)	0.837
Parity = 0	−1.35 (−1.59, −1.10)	−1.80 (−2.07, −1.53)	−1.87 (−2.16, −1.58)		0.52 (0.17, 0.88)	0.004	0.07 (−0.30, 0.44)	0.710	0.45 (0.11, 0.79)	0.009

Regression model controls for site, study arm, parity, age <20 years, anemia (hemoglobin <12 g/dL), low body mass index (BMI <18.5 kg/m^2^), no formal education, newborn sex, and low household socioeconomic status (SES). The SES tally provides the number of indicators available from the following list: electricity, improved water source, sanitation, man-made flooring, improved cooking fuels, and household assets. Low SES is defined as having ≤2 SES indicators present. CI, confidence interval.

**Table 4:** Regression results of neonatal anthropometry by parity: individual site data.

Outcome	Arm 1	Arm 2	Arm 3	Parity x Arm	Arm 1 vs. Arm 3		Arm 2 vs. Arm 3		Arm 1 vs. Arm 2	
	Adjusted Mean (95% CI)	Adjusted Mean (95% CI)	Adjusted Mean (95% CI)	Interaction (*p*-Value)	Adjusted Mean Difference (95% CI)	*p*-Value	Adjusted Mean Difference (95% CI)	*p*-Value	Adjusted Mean Difference (95% CI)	*p*-Value
**Guatemala**										
**Length-for-age *z*-score**										
Parity ≥1	−0.84 (−1.03, −0.66)	−0.67 (−0.85, −0.49)	−0.90 (−1.08, −0.71)	0.219	0.05 (−0.14, 0.25)	0.598	0.23 (0.03, 0.42)	0.022	−0.17 (−0.37, 0.02)	0.078
Parity = 0	−0.92 (−1.43, −0.42)	−1.27 (−1.79, −0.74)	−0.84 (−1.46, −0.23)		−0.08 (−0.85, 0.69)	0.843	−0.42 (−1.21, 0.36)	0.290	0.35 (−0.35, 1.04)	0.330
**Weight-for-age *z*-score**										
Parity ≥1	−0.71 (−0.86, −0.56)	−0.61 (−0.75, −0.46)	−0.81 (−0.97, −0.66)	0.420	0.10 (−0.08, 0.28)	0.279	0.21 (0.03, 0.38)	0.021	−0.11 (−0.28, 0.07)	0.228
Parity = 0	−0.91 (−1.36, −0.45)	−1.04 (−1.51, −0.56)	−0.75 (−1.31, −0.20)		−0.15 (−0.85, 0.55)	0.666	−0.28 (−0.99, 0.43)	0.434	0.13 (−0.51, 0.77)	0.688
**Weight to length ratio-for-age *z*-score**										
Parity ≥1	−0.84 (−1.06, −0.63)	−0.72 (−0.93, −0.51)	−1.00 (−1.23, −0.78)	0.518	0.16 (−0.10, 0.42)	0.228	0.28 (0.02, 0.54)	0.032	−0.12 (−0.38, 0.14)	0.358
Parity = 0	−1.17 (−1.83, −0.51)	−1.24 (−1.93, −0.55)	−0.91 (−1.71, −0.10)		−0.26 (−1.28, 0.75)	0.613	−0.34 (−1.37, 0.70)	0.522	0.08 (−0.85, 1.00)	0.872
**India**										
**Length-for-age *z*-score**										
Parity ≥1	−0.63 (−0.86, −0.40)	−0.57 (−0.79, −0.36)	−0.79 (−1.03, −0.55)	0.270	0.17 (−0.08, 0.42)	0.190	0.22 (−0.02, 0.46)	0.075	−0.05 (−0.29, 0.19)	0.678
Parity = 0	−0.73 (−1.04, −0.43)	−0.99 (−1.33, −0.65)	−1.27 (−1.62, −0.91)		0.53 (0.11, 0.96)	0.013	0.27 (−0.17, 0.71)	0.224	0.26 (−0.14, 0.67)	0.203
**Weight-for-age *z*-score**										
Parity ≥1	−1.12 (−1.32, −0.92)	−1.14 (−1.33, −0.95)	−1.30 (−1.50, −1.09)	0.101	0.18 (−0.05, 0.41)	0.125	0.15 (−0.06, 0.37)	0.167	0.02 (−0.20, 0.24)	0.829
Parity = 0	−1.14 (−1.40, −0.87)	−1.47 (−1.77, −1.17)	−1.79 (−2.11, −1.47)		0.65 (0.27, 1.04)	0.001	0.32 (−0.08, 0.72)	0.116	0.33 (−0.03, 0.70)	0.073
**Weight to length ratio-for-age *z*-score**										
Parity ≥1	−1.68 (−1.96, −1.40)	−1.71 (−1.97, −1.45)	−1.90 (−2.19, −1.62)	0.041	0.23 (−0.09, 0.55)	0.167	0.20 (−0.11, 0.50)	0.210	0.03 (−0.28, 0.34)	0.853
Parity = 0	−1.60 (−1.98, −1.22)	−2.19 (−2.61, −1.77)	−2.59 (−3.04, −2.14)		0.99 (0.45, 1.54)	0.000	0.40 (−0.17, 0.96)	0.167	0.60 (0.08, 1.11)	0.023
**Pakistan**										
**Length-for-age *z*-score**										
Parity ≥1	−0.46 (−0.71, −0.22)	−0.69 (−0.92, −0.45)	−0.69 (−0.94, −0.44)	0.037	0.22 (−0.09, 0.54)	0.167	0.00 (−0.31, 0.31)	0.991	0.22 (−0.09, 0.53)	0.158
Parity = 0	−0.49 (−0.82, −0.17)	−0.53 (−0.90, −0.16)	−1.27 (−1.64, −0.89)		0.77 (0.31, 1.24)	0.001	0.74 (0.24, 1.23)	0.003	0.04 (−0.42, 0.49)	0.875
**Weight-for-age *z*-score**										
Parity ≥1	−0.68 (−0.88, −0.47)	−0.84 (−1.03, −0.64)	−0.81 (−1.02, −0.61)	0.500	0.13 (−0.14, 0.41)	0.334	−0.03 (−0.29, 0.24)	0.850	0.16 (−0.10, 0.42)	0.236
Parity = 0	−0.90 (−1.17, −0.63)	−1.24 (−1.55, −0.93)	−1.31 (−1.63, −1.00)		0.41 (0.02, 0.81)	0.041	0.07 (−0.35, 0.49)	0.740	0.34 (−0.05, 0.73)	0.084
**Weight to length ratio-for-age *z*-score**										
Parity ≥1	−0.88 (−1.15, −0.60)	−1.08 (−1.34, −0.82)	−1.08 (−1.35, −0.80)	0.718	0.20 (−0.17, 0.57)	0.291	−0.01 (−0.37, 0.35)	0.967	0.21 (−0.15, 0.57)	0.258
Parity = 0	−1.39 (−1.76, −1.03)	−1.85 (−2.27, −1.43)	−1.64 (−2.07, −1.21)		0.24 (−0.30, 0.79)	0.374	−0.21 (−0.79, 0.36)	0.465	0.46 (−0.07, 0.99)	0.088

Regression model controls for study arm, parity, age <20 years, anemia (hemoglobin <12), low body mass index (BMI, <18.5 kg/m^2^), no formal education, newborn sex, and low household socioeconomic status (SES). The SES tally provides the number of indicators available from the following list: electricity, improved water source, sanitation, man-made flooring, improved cooking fuels, and household assets. Low SES is defined as having ≤2 SES indicators present. CI, confidence interval.

**Table 5:** Regression results of neonatal anthropometry by anemia: combined sites.

Outcome	Arm 1	Arm 2	Arm 3	Anemia x Arm Interaction (*p*-Value)	Arm 1 vs. Arm 3		Arm 2 vs. Arm 3		Arm 1 vs. Arm 2	
	Adjusted Mean (95% CI)	Adjusted Mean (95% CI)	Adjusted Mean (95% CI)		Adjusted Mean Difference (95% CI)	*p*-Value	Adjusted Mean Difference (95% CI)	*p*-Value	Adjusted Mean Difference (95% CI)	*p*-Value
**Length-for-age *z*-score**										
-Anemia	−0.76 (−0.92, −0.60)	−0.73 (−0.89, −0.57)	−0.79 (−0.95, −0.63)	0.037	0.03 (−0.17, 0.23)	0.759	0.06 (−0.13, 0.26)	0.535	−0.03 (−0.23, 0.17)	0.758
+Anemia	−0.72 (−0.86, −0.58)	−0.77 (−0.91, −0.63)	−1.08 (−1.23, −0.93)		0.36 (0.19, 0.53)	<0.001	0.31 (0.15, 0.48)	<0.001	0.05 (−0.11, 0.21)	0.564
**Weight-for-age *z*-score**										
-Anemia	−0.96 (−1.10, −0.82)	−0.91 (−1.04, −0.77)	−0.94 (−1.08, −0.80)	0.007	−0.02 (−0.20, 0.16)	0.846	0.03 (−0.14, 0.21)	0.699	−0.05 (−0.23, 0.12)	0.559
+Anemia	−0.97 (−1.08, −0.85)	−1.13 (−1.24, −1.01)	−1.32 (−1.45, −1.20)		0.36 (0.21, 0.51)	<0.001	0.20 (0.05, 0.34)	0.010	0.16 (0.02, 0.31)	0.028
**Weight to length ratio-for-age *z*-score**										
-Anemia	−1.33 (−1.53, −1.13)	−1.26 (−1.46, −1.07)	−1.30 (−1.50, −1.10)	0.009	−0.03 (−0.29, 0.22)	0.785	0.03 (−0.21, 0.28)	0.790	−0.07 (−0.31, 0.18)	0.585
+Anemia	−1.30 (−1.47, −1.13)	−1.54 (−1.71, −1.36)	−1.77 (−1.95, −1.59)		0.47 (0.26, 0.68)	<0.001	0.23 (0.02, 0.44)	0.029	0.24 (0.04, 0.44)	0.022

Regression model controls for site, study arm, parity, age <20 years, anemia (hemoglobin (Hb) <12 g/dL), low body mass index (BMI, <18.5 kg/m^2^), no formal education, newborn sex, and low household socioeconomic status (SES). The SES tally provides the number of indicators available from the following list: electricity, improved water source, sanitation, man-made flooring, improved cooking fuels, and household assets. Low SES is defined as having ≤2 SES indicators present. -Anemia includes participants with Hb ≥12 g/dL; +Anemia includes participants with Hb <12 g/dL. CI, confidence interval.

**Table 6:** Regression results of neonatal anthropometry by anemia: individual site data.

	Arm 1	Arm 2	Arm 3	Anemia x Arm	Arm 1 vs. Arm 3		Arm 2 vs. Arm 3		Arm 1 vs. Arm 2	
	Adjusted Mean (95% CI)	Adjusted Mean (95% CI)	Adjusted Mean (95% CI)	Interaction *p*-Value	Adjusted Mean Difference (95% CI)	*p*-Value	Adjusted Mean Difference (95% CI)	*p*-Value	Adjusted Mean Difference (95% CI)	*p*-Value
**Guatemala**										
**Length-for-age *z*-score**										
-Anemia	−0.95 (−1.17, −0.74)	−0.74 (−0.96, −0.53)	−0.92 (−1.13, −0.70)	0.042	−0.04 (−0.24, 0.16)	0.716	0.17 (−0.03, 0.37)	0.089	−0.21 (−0.41, −0.01)	0.040
+Anemia	−0.67 (−1.06, −0.27)	−0.99 (−1.39, −0.58)	−1.35 (−1.81, −0.89)		0.68 (0.12, 1.24)	0.018	0.36 (−0.20, 0.92)	0.209	0.32 (−0.19, 0.83)	0.218
**Weight-for-age *z*-score**										
-Anemia	−0.87 (−1.05, −0.69)	−0.72 (−0.89, −0.54)	−0.87 (−1.06, −0.69)	0.022	−0.00 (−0.19, 0.18)	0.985	0.15 (−0.03, 0.33)	0.092	−0.16 (−0.34, 0.02)	0.091
+Anemia	−0.50 (−0.84, −0.15)	−0.80 (−1.15, −0.45)	−1.24 (−1.65, −0.82)		0.74 (0.23, 1.25)	0.005	0.44 (−0.07, 0.95)	0.093	0.30 (−0.16, 0.76)	0.196
**Weight to length ratio-for age *z*-score**										
-Anemia	−1.09 (−1.35, −0.83)	−0.91 (−1.17, −0.65)	−1.11 (−1.38, −0.85)	0.050	0.02 (−0.25, 0.29)	0.870	0.20 (−0.06, 0.47)	0.126	−0.18 (−0.45, 0.08)	0.176
+Anemia	−0.55 (−1.06, −0.04)	−0.90 (−1.41, −0.39)	−1.55 (−2.15, −0.95)		1.00 (0.26, 1.75)	0.008	0.65 (−0.09, 1.39)	0.085	0.35 (−0.32, 1.02)	0.306
**India**										
**Length-for-age *z*-score**										
-Anemia	−0.63 (−1.12, −0.15)	−0.84 (−1.33, −0.35)	−1.08 (−1.70, −0.45)	0.842	0.44 (−0.34, 1.22)	0.265	0.24 (−0.54, 1.02)	0.554	0.21 (−0.46, 0.88)	0.545
+Anemia	−0.71 (−0.89, −0.54)	−0.73 (−0.90, −0.56)	−0.96 (−1.15, −0.78)		0.25 (0.03, 0.48)	0.028	0.23 (0.01, 0.45)	0.037	0.02 (−0.20, 0.24)	0.868
**Weight-for-age *z*-score**										
-Anemia	−1.30 (−1.73, −0.87)	−1.26 (−1.70, −0.83)	−1.41 (−1.97, −0.85)	0.821	0.11 (−0.60, 0.82)	0.755	0.15 (−0.56, 0.86)	0.687	−0.03 (−0.64, 0.58)	0.916
+Anemia	−1.14 (−1.29, −1.00)	−1.27 (−1.42, −1.13)	−1.47 (−1.62, −1.31)		0.32 (0.12, 0.53)	0.002	0.20 (−0.01, 0.40)	0.056	0.13 (−0.07, 0.33)	0.203
**Weight to length ratio-for age *z*-score**										
-Anemia	−1.99 (−2.59, −1.39)	−1.85 (−2.46, −1.24)	−1.98 (−2.76, −1.19)	0.592	−0.02 (−1.01, 0.98)	0.975	0.12 (−0.87, 1.12)	0.805	−0.14 (−0.99, 0.71)	0.746
+Anemia	−1.65 (−1.86, −1.45)	−1.88 (−2.08, −1.67)	−2.12 (−2.34, −1.90)		0.47 (0.18, 0.76)	0.001	0.25 (−0.03, 0.53)	0.085	0.22 (−0.06, 0.50)	0.117
**Pakistan**										
**Length-for-age *z*-score**										
-Anemia	−0.29 (−0.65, 0.07)	−0.76 (−1.12, −0.41)	−0.43 (−0.78, −0.08)	0.032	0.14 (−0.35, 0.63)	0.575	−0.33 (−0.81, 0.15)	0.177	0.47 (−0.02, 0.97)	0.061
+Anemia	−0.69 (−0.93, −0.46)	−0.76 (−0.99, −0.52)	−1.19 (−1.45, −0.94)		0.50 (0.19, 0.81)	0.002	0.44 (0.12, 0.75)	0.006	0.06 (−0.24, 0.36)	0.676
**Weight-for-age *z*-score**										
-Anemia	−0.77 (−1.08, −0.47)	−1.06 (−1.36, −0.77)	−0.70 (−1.00, −0.41)	0.088	−0.07 (−0.49, 0.35)	0.746	−0.36 (−0.77, 0.05)	0.085	0.29 (−0.13, 0.71)	0.175
+Anemia	−0.89 (−1.08, −0.70)	−1.08 (−1.28, −0.89)	−1.24 (−1.45, −1.04)		0.35 (0.09, 0.61)	0.010	0.16 (−0.11, 0.43)	0.240	0.19 (−0.06, 0.45)	0.143
**Weight to length ratio-for age *z*-score**										
-Anemia	−1.21 (−1.61, −0.80)	−1.52 (−1.92, −1.13)	−1.00 (−1.39, −0.60)	0.095	−0.21 (−0.78, 0.36)	0.468	−0.53 (−1.09, 0.03)	0.063	0.32 (−0.25, 0.89)	0.270
+Anemia	−1.17 (−1.42, −0.91)	−1.43 (−1.69, −1.18)	−1.57 (−1.85, −1.30)		0.40 (0.04, 0.76)	0.029	0.14 (−0.23, 0.50)	0.458	0.27 (−0.08, 0.61)	0.134

Regression model controls for study arm, parity, age < 20 years, anemia (hemoglobin (Hb) <12), low body mass index (BMI, <18.5 kg/m^2^), no formal education, newborn sex, and low household socioeconomic status (SES). The SES tally provides the number of indicators available from the following list: electricity, improved water source, sanitation, man-made flooring, improved cooking fuels, and household assets. Low SES is defined as having ≤2 SES indicators present. -Anemia includes participants with Hb ≥12 g/dL; +Anemia includes participants with Hb <12 g/dL. CI, confidence interval.

## Supplementary Materials

The following are available online at https://www.mdpi.com/2072-6643/11/10/2534/s1: Figure S1: CONSORT diagram for Women First Maternal Preconception Nutrition Trial, including participants from Guatemala, India, and Pakistan. Overall screening, random assignment, gestational age availability and obtainment of primary outcome by treatment arm. ^1^ Percentage of those randomly assigned. Excludes women who became pregnant <3 months into the study. The women who had eligible pregnancies may have had delivery data obtained or they may have exited the study before delivery. ^2^ GA at birth is defined as the age at the time of the ultrasound based on the ultrasound plus time until birth if the ultrasound was done between 6 weeks + 0 day and 13 weeks + 6 days and the GA at birth was between 24 weeks + 0 day and 42 weeks + 6 days. If the ultrasound was not conducted during the GA previously mentioned, then the GA at birth is missing. ^3^ Primary outcome was obtained for live newborns with 3 length measurements taken within 48 h of delivery. Among women, primary outcome obtained from ≥1 infants of the woman. CONSORT, Consolidated Standards of Reporting Trials; GA, gestational age; MTP, medical termination of pregnancy.; Table S1: Descriptive subgroup analysis by parity and body mass index (BMI); Table S2: Regression results of neonatal anthropometry by newborn sex: combined sites.
