# Supplementary material for: Maternal Characteristics Affect Fetal Growth Response in the Women First Preconception Nutrition Trial
Source: Nutrients. 2019 Oct 21;11(10):2534. doi: 10.3390/nu11102534 (PMC6835723; doi:10.3390/nu11102534)
Supplement: Supplementary file 1 [file nutrients-11-02534-s001.pdf]

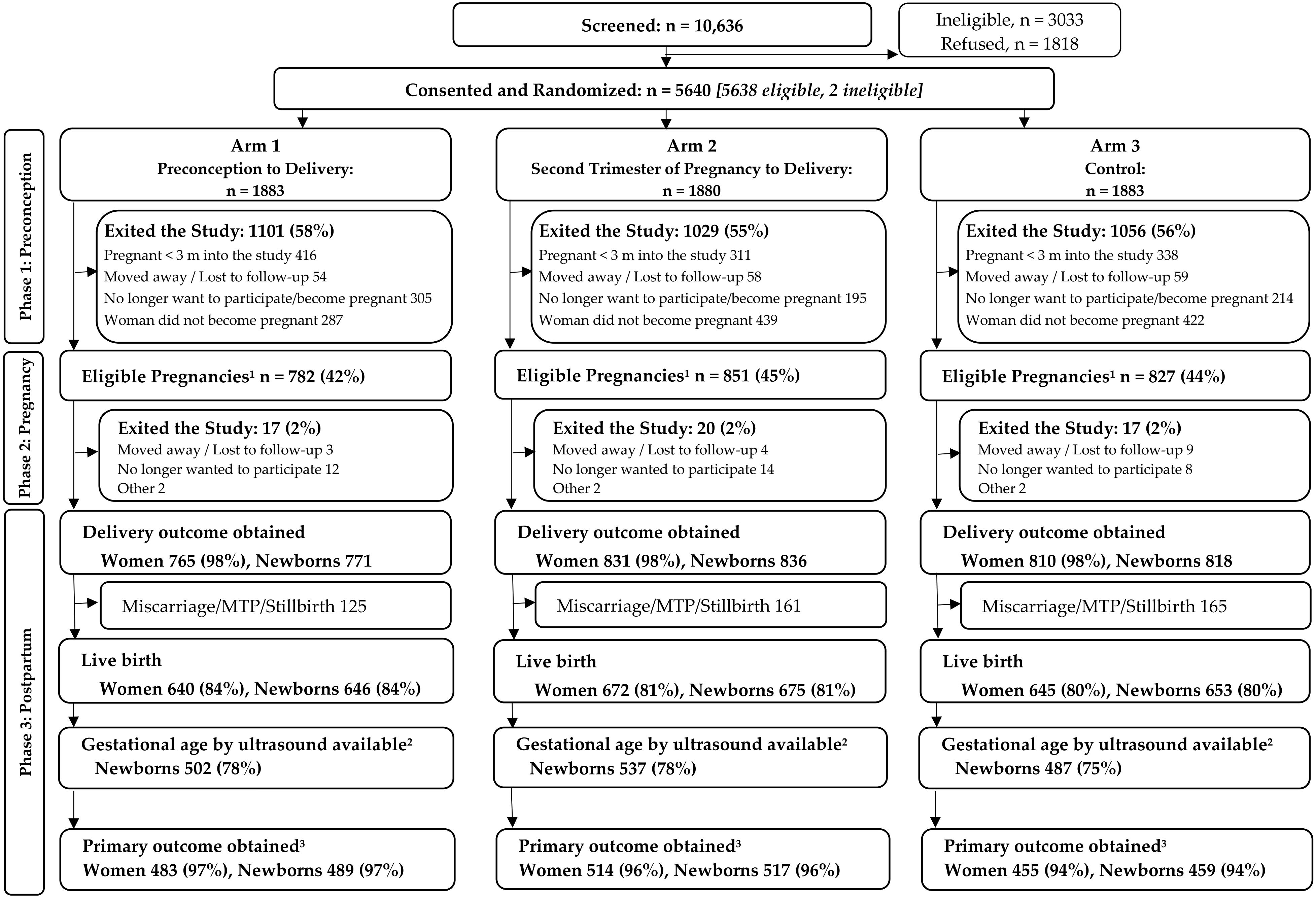
Screened: n = 10,636
Ineligible, n = 3033
Refused, n = 1818
Consented and Randomized: n = 5640 [5638 eligible, 2 ineligible]
Phase 1: Preconception
Phase 2: Pregnancy
Phase 3: Postpartum
Arm 1
Preconception to Delivery:
n = 1883
Arm 2
Second Trimester of Pregnancy to Delivery:
n = 1880
Arm 3
Control:
n = 1883
Exited the Study: 1101 (58%)
Pregnant < 3 m into the study 416
Moved away / Lost to follow-up 54
No longer want to participate/become pregnant 305
Woman did not become pregnant 287
Exited the Study: 1029 (55%)
Pregnant < 3 m into the study 311
Moved away / Lost to follow-up 58
No longer want to participate/become pregnant 195
Woman did not become pregnant 439
Exited the Study: 1056 (56%)
Pregnant < 3 m into the study 338
Moved away / Lost to follow-up 59
No longer want to participate/become pregnant 214
Woman did not become pregnant 422
Eligible Pregnancies[1] n = 782 (42%)
Eligible Pregnancies[1] n = 851 (45%)
Eligible Pregnancies[1] n = 827 (44%)
Exited the Study: 17 (2%)
Moved away / Lost to follow-up 3
No longer wanted to participate 12
Other 2
Exited the Study: 20 (2%)
Moved away / Lost to follow-up 4
No longer wanted to participate 14
Other 2
Exited the Study: 17 (2%)
Moved away / Lost to follow-up 9
No longer wanted to participate 8
Other 2
Delivery outcome obtained
Women 765 (98%), Newborns 771
Delivery outcome obtained
Women 831 (98%), Newborns 836
Delivery outcome obtained
Women 810 (98%), Newborns 818
Miscarriage/MTP/Stillbirth 125
Miscarriage/MTP/Stillbirth 161
Miscarriage/MTP/Stillbirth 165
Live birth
Women 640 (84%), Newborns 646 (84%)
Live birth
Women 672 (81%), Newborns 675 (81%)
Live birth
Women 645 (80%), Newborns 653 (80%)
Gestational age by ultrasound available[2]
Newborns 502 (78%)
Gestational age by ultrasound available[2]
Newborns 537 (78%)
Gestational age by ultrasound available[2]
Newborns 487 (75%)
Primary outcome obtained[3]
Women 483 (97%), Newborns 489 (97%)
Primary outcome obtained[3]
Women 514 (96%), Newborns 517 (96%)
Primary outcome obtained[3]
Women 455 (94%), Newborns 459 (94%)

Supplementary Figure 1. CONSORT diagram for Women First Maternal Preconception Nutrition Trial, including participants from Guatemala, India, and Pakistan. Overall screening, random assignment, gestational age availability and obtainment of primary outcome by treatment arm.

[1]Percentage of those randomly assigned. Excludes women who became pregnant <3 mo into the study. The women who had eligible pregnancies may have had delivery data obtained or they may have exited the study before delivery.

[2]GA at birth is defined as the age at the time of the ultrasound based on the ultrasound plus time until birth if the ultrasound was done between 6 wk + 0 d and 13 wk + 6 d and the GA at birth was between 24 wk + 0 d and 42 wk + 6 d. If the ultrasound was not conducted during the GA previously mentioned, then the GA at birth is missing.

[3]Primary outcome was obtained for live newborns with 3 length measurements taken within 48 h of delivery. Among women, primary outcome obtained from ≥ 1 infants of the woman.

CONSORT, Consolidated Standards of Reporting Trials; GA, gestational age; MTP, medical termination of pregnancy.

Online Supporting Material

Supplemental Table 1: Descriptive subgroup analysis by parity and body mass index (BMI)

**Outcome**	**Arm 1**		**Arm 2**		**Arm 3**		**Arm 1 vs. 3**	**Arm 2 vs. 3**	**Arm 1 vs. 2**
Subgroup	**N**	**Mean ± SD**	**N**	**Mean ± SD**	**N**	**Mean ± SD**	***P*-value**	***P*-value**	***P*-value**
**Length-for-age Z-score**									
Parity = 0: Normal/High BMI	77	-0.74 ± 1.06	63	-0.82 ± 1.20	56	-1.11 ± 0.99	0.057	0.149	0.680
Parity = 0: Low BMI[2]	46	-0.68 ± 1.00	34	-1.00 ± 1.15	28	-1.62 ± 1.19	< 0.001	0.027	0.205
Parity ≥ 0: Normal/High BMI	287	-0.64 ± 0.97	323	-0.60 ± 1.00	290	-0.77 ± 0.98	0.111	0.027	0.566
Parity ≥ 0: Low BMI	79	-0.81 ± 0.86	95	-0.82 ± 1.09	85	-0.87 ± 1.09	0.699	0.751	0.931
**Weight-for-age Z-score**									
Parity = 0: Normal/High BMI	77	-1.01 ± 1.00	63	-1.24 ± 1.07	56	-1.33 ± 0.93	0.077	0.647	0.181
Parity = 0: Low BMI	46	-1.16 ± 1.00	34	-1.63 ± 0.89	28	-1.85 ± 0.80	0.002	0.345	0.026
Parity ≥ 0: Normal/High BMI	287	-0.77 ± 0.89	323	-0.75 ± 0.90	290	-0.90 ± 0.83	0.064	0.029	0.779
Parity ≥ 0: Low BMI	79	-1.12 ± 0.88	96	-1.21 ± 0.76	85	-1.16 ± 1.09	0.804	0.701	0.528
**Weight to length ratio-for age Z-score**									
Parity = 0: Normal/High BMI	77	-1.45 ± 1.47	62	-1.74 ± 1.39	55	-1.81 ± 1.41	0.159	0.796	0.239
Parity = 0: Low BMI	44	-1.62 ± 1.37	34	-2.44 ± 1.20	27	-2.43 ± 1.25	0.011	0.979	0.006
Parity ≥ 0: Normal/High BMI	286	-1.03 ± 1.29	321	-0.98 ± 1.25	288	-1.20 ± 1.14	0.096	0.033	0.676
Parity ≥ 0: Low BMI	77	-1.51 ± 1.24	96	-1.73 ± 1.10	85	-1.65 ± 1.56	0.493	0.690	0.275

For each subgroup, treatment arms were compared within a linear regression model, using contrast statements.

[1]Low BMI defined as < 18.5 kg/m^2.

Online Supporting Material

Supplemental Table 2. Regression results of neonatal anthropometry by newborn sex: combined sites

Outcome	Arm 1 Adjusted Mean (95% CI)	Arm 2 Adjusted Mean (95% CI)	Arm 3 Adjusted Mean (95% CI)	Sex x Arm Interaction *P*-value	Arm 1 v Arm 3 Adjusted Mean Difference (95% CI)	Arm 1 v Arm 3 *P*-Value	Arm 2 v Arm 3 Adjusted Mean Difference (95% CI)	Arm 2 v Arm 3 *P*-Value	Arm 1 v Arm 2 Adjusted Mean Difference (95% CI)	Arm 1 v Arm 2 *P*-Value
**Length-for-age Z-score**										
Female	-0.78 (-0.92, -0.63)	-0.88 (-1.02, -0.74)	-0.95 (-1.10, -0.80)	0.071	0.17 (-0.01, 0.35)	0.066	0.06 (-0.12, 0.24)	0.498	0.11 (-0.07, 0.28)	0.223
Male	-0.67 (-0.82, -0.53)	-0.60 (-0.74, -0.45)	-0.95 (-1.10, -0.80)		0.28 (0.09, 0.46)	0.003	0.35 (0.17, 0.54)	< 0.001	-0.08 (-0.26, 0.10)	0.386
**Weight-for-age Z-score**										
Female	-0.98 (-1.10, -0.85)	-1.14 (-1.26, -1.02)	-1.12 (-1.25, -0.99)	0.028	0.14 (-0.02, 0.30)	0.086	-0.03 (-0.18, 0.13)	0.757	0.17 (0.01, 0.32)	0.035
Male	-0.91 (-1.03, -0.78)	-0.89 (-1.01, -0.76)	-1.17 (-1.30, -1.04)		0.26 (0.10, 0.43)	0.002	0.28 (0.12, 0.44)	0.001	-0.02 (-0.18, 0.14)	0.815
**Weight to length ratio-for age Z-score**										
Female	-1.33 (-1.50, -1.15)	-1.56 (-1.73, -1.38)	-1.51 (-1.70, -1.32)	0.052	0.18 (-0.05, 0.41)	0.117	-0.05 (-0.27, 0.18)	0.678	0.23 (0.01, 0.45)	0.039
Male	-1.25 (-1.43, -1.07)	-1.24 (-1.42, -1.06)	-1.59 (-1.78, -1.40)		0.34 (0.11, 0.57)	0.004	0.34 (0.12, 0.57)	0.003	-0.01 (-0.23, 0.22)	0.951

Regression model controls for site, study arm, parity, age < 20 years, anemia (hemoglobin < 12 mg/dL), low BMI (< 18.5 kg/m^2), no formal education, newborn sex, and low household socioeconomic status (SES). The SES tally provides the number of indicators available from the following list: electricity, improved water source, sanitation, man-made flooring, improved cooking fuels, and household assets. Low SES is defined as having ≤ 2 SES indicators present. CI, confidence interval.
